# Changes in aortic diameter induced by weight loss: The HELENA trial- whole-body MR imaging in a dietary intervention trial

**DOI:** 10.3389/fphys.2022.976949

**Published:** 2022-09-20

**Authors:** Sibylle Stoll, Solomon A. Sowah, Matthias A. Fink, Tobias Nonnenmacher, Mirja E. Graf, Theron Johnson, Christopher L. Schlett, Oyunbileg von Stackelberg, Romy Kirsten, Fabian Bamberg, Jeffrey Keller, Cornelia M. Ulrich, Rudolf Kaaks, Hans-Ulrich Kauczor, Fabian Rengier, Tilman Kühn, Johanna Nattenmüller

**Affiliations:** ^1^ Heidelberg University Hospital, Diagnostic and Interventional Radiology, Heidelberg, Germany; ^2^ German Cancer Research Center (DKFZ), Division of Cancer Epidemiology, Heidelberg, Germany; ^3^ Department of Diagnostic and Interventional Radiology, Medical Center - Faculty of Medicine, University of Freiburg, Freiburg, Germany; ^4^ National Center for Tumor Diseases (NCT), Liquid Biobank, Heidelberg, Germany; ^5^ Division of Preventive Oncology, German Cancer Research Center (DKFZ), Heidelberg, Germany; ^6^ Federal Office of Public Health, Bern, Switzerland; ^7^ Huntsman Cancer Institute and Department of Population Health Sciences, University of Utah, Salt Lake City, UT, United States

**Keywords:** aortic diameter, magnetic resonance imaging, obesity, overweight, calorie restriction weight loss, aortic aneurysm

## Abstract

Obesity-related metabolic disorders such as hypertension, hyperlipidemia and chronic inflammation have been associated with aortic dilatation and resulting in aortic aneurysms in many cases. Whether weight loss may reduce the risk of aortic dilatation is not clear. In this study, the diameter of the descending thoracic aorta, infrarenal abdominal aorta and aortic bifurcation of 144 overweight or obese non-smoking adults were measured by MR-imaging, at baseline, and 12 and 50 weeks after weight loss by calorie restriction. Changes in aortic diameter, anthropometric measures and body composition and metabolic markers were evaluated using linear mixed models. The association of the aortic diameters with the aforementioned clinical parameters was analyzed using Spearman`s correlation. Weight loss was associated with a reduction in the thoracic and abdominal aortic diameters 12 weeks after weight loss (predicted relative differences for Quartile 4: 2.5% ± 0.5 and -2.2% ± 0.8, *p* < 0.031; respectively). Furthermore, there was a nominal reduction in aortic diameters during the 50-weeks follow-up period. Aortic diameters were positively associated with weight, visceral adipose tissue, glucose, HbA1c and with both systolic and diastolic blood pressure. Weight loss induced by calorie restriction may reduce aortic diameters. Future studies are needed to investigate, whether the reduction of aortic diameters via calorie restriction may help to prevent aortic aneurysms.

## Introduction

The prevalence of obesity is increasing worldwide and has tripled since 1975 (World Health Organisation (WHO), 2021). Sequelae of obesity are an increased risk for the metabolic syndrome (Engin, 2017), cardiovascular diseases (Alpert et al., 2016), and several cancer types (Avgerinos et al., 2019). Thus, obesity causes worldwide high costs in the economy and health care systems and therefore it is a crucial interest of public health to prevent obesity and its complications (Tremmel et al., 2017; Hamilton et al., 2018; De Lorenzo et al., 2020).

Aortic aneurysm, which is defined as a dilatation of the aorta to greater than 1.5 times normal size, has a relevant global death rate of 2.8 per 100,000 population in 2010, which is further increasing according to the global burden of disease study in comparison to 1990 (2.5) (Sampson et al., 2014). For the development of an aortic aneurysm several risk factors are described in literature: smoking, family history, age, male gender, hypertension, cardiovascular and atherosclerotic diseases (Lederle et al., 2000; Allison et al., 2008; Kent et al., 2010). As several secondary diseases of obesity are also main risk factors for aortic aneurysm, it is not surprising that obesity itself is also associated with aortic aneurysm, despite not being listed as first line risk factor (Cronin et al., 2013). There are several studies which describe an association of markers of obesity with the presence of an aortic aneurysm or with increasing aortic diameter (Allison et al., 2008; Gorter et al., 2008; Kent et al., 2010; Long et al., 2010; Cronin et al., 2013). These studies used either waist circumference (Lederle et al., 2000; Golledge et al., 2007; Gorter et al., 2008) or BMI as measure of obesity (Lederle et al., 2000; Allison et al., 2008; Kent et al., 2010), whereas waist circumference as indicator for central obesity seems to be more strongly associated with aortic aneurysm than BMI (Lederle et al., 2000).

The pathogenesis of an aortic aneurysm is not fully understood (Police et al., 2009; Police et al., 2010). However, degradation of the aortic elastin, inflammatory processes and neovascularization of the adventitia seem to be crucial steps towards the formation of an aortic aneurism (Police et al., 2009; Police et al., 2010). In this process, also obesity with its altered metabolic state could contribute (Henrichot et al., 2005; Police et al., 2009). Obesity is known to cause a pro-inflammatory state with an imbalance of inflammatory chemokines like interleukin 6, interleukin eight or TNF-alpha, which are secreted by adipose tissue, which surrounds directly the aorta and other great vessels (Henrichot et al., 2005; Police et al., 2009). Again, inflammation is thought to weaken and dilate vessels, which may explain the contribution of obesity (Choke et al., 2005; Nordon et al., 2009; Cronin et al., 2013).

A number of risk factors for aortic aneurysm are known to improve after weight loss. There are several studies which report a lowering of blood pressure or reduction of anti-hypertensive medications after weight-loss (Stevens et al., 1993; Jones et al., 1999; Sjostrom et al., 1999; Moore et al., 2005; Fantin et al., 2019; Ortiz-Gomez et al., 2020). Also, cardiovascular diseases are reported to improve after weight loss (Weiss et al., 2016; Ma et al., 2017; Clifton, 2018). Additionally, chronic inflammation as central part in the pathogenesis of the aortic aneurysm seems to be reduced by weight loss via a reduction of pro-inflammatory cytokines and increase of anti-inflammatory chemokines (You and Nicklas, 2006). As several sequelae of obesity improve after weight loss, it is conceivable that the aortic diameter and thus the risk of an aortic aneurysm can equally be reduced after weight loss. To the best of our knowledge, no dedicated human study has analyzed the effect of weight loss on aortic aneurysm or aortic diameter. One study on Angiotensin II-induced aortic aneurysm in mice found that weight loss limits the expansion and neovascularization of the aortic adventitia, which has been suggested to be associated with a favorable effect of weight loss on aortic aneurysm progression (Police et al., 2010).

The purpose of this study was to investigate the effect of diet-induced moderate weight loss on the diameter of the abdominal aorta as measured by MRI as proxy for aortic aneurysm and prevention of aortic aneurysm in overweight or obese non-smoking, healthy individuals over 1 year. Additionally, we studied the association of the aortic diameter with MR-based body fat depots like the visceral adipose tissue (VAT), the subcutaneous adipose tissue (SAT) and liver fat content (LFC), anthropometric measurements, blood pressure as well as metabolic biomarkers. Our hypothesis was that a weight loss intervention is associated with decreases in aortic dimensions, which are correlated with body fat depots and other anthropometric measurements.

## Materials and methods

### Study design and study population

Imaging and clinical data from the HELENA Trial (NCT02449148, clinicaltrials.gov), which initially examined the effects of intermittent calorie restriction (ICR) versus continuous calorie restriction (CCR), were used for the present post hoc analysis. The study was conducted from May 2015 to May 2017 at the German Cancer Research Center (DKFZ) and the University Hospital Heidelberg. The study was approved by the ethics committee in Heidelberg, Germany. All participants provided written informed consent. Details of the study design, participant recruitment and data collection have been published previously (Schubel et al., 2016; Schubel et al., 2018).

In short, the study population included 150 overweight or obese non-smokers (50% male) aged between 35–65 years without any history of diabetes, major cardiovascular disease, cancer or other severe organ dysfunctions/chronic diseases. Further exclusion criteria were HbA1c levels ≥6.5% as well as fasting plasma glucose levels >126 mg/dl. The participants were randomly assigned into one of three study groups i.e., intermittent calorie restriction (ICR), continuous calorie restriction (CCR) or control group. The ICR group participants were advised to reduce the calorie intake by ∼75% on two non-consecutive days per week whereas the CCR group participants were advised to reduce the calorie intake by ∼20% each day. Participants assigned to the control group were not advised to reduce their calorie intake. All study participants were advised to adhere to the dietary recommendations of the German Nutrition Society (Jungvogel et al., 2013; Oberritter et al., 2013). The study consisted of a 12-weeks intervention phase, followed by a 12-weeks maintenance phase and a 26-weeks follow-up phase. Anthropometric assessments, clinical examinations, questionnaires and blood biomarker assessments were performed across the following study time points i.e. baseline (T0), week 12 (T1), week 24 (T2) and week 50 (T3). Each study participant was given a phone call every 2 weeks by dietitians during intervention phase for monitoring of possible side effects. No major side effects were documented during the trial. All study participants provided written consent prior to study enrolment.

MRI examinations were conducted for every participant at three time-points: at baseline, after 12 weeks of intervention, and after 50 weeks. Defined exclusion criteria for the MRI examinations served cardiac pacemakers and defibrillators, non-removable, medical and/or electronic foreign bodies and joint prostheses without approval for 1.5 T-MR, metallic foreign bodies and claustrophobia. From the original 150 study participants, a total of 144 (96.0%), 143 (95.3), and 136 (90.7%) participants completed the intervention phase, maintenance phase and follow-up phase respectively. We finally included 144 participants in the present post-hoc analysis.

### Laboratory analyses

Blood samples of each participant were obtained at baseline, after 12 weeks of intervention and after follow-up phase was finished (50 weeks total). Routine blood parameters such as ALT (alanine aminotransferase), AST (aspartate aminotransferase), GGT (gamma-glutamyltransferase), insulin, glucose, HDL (high density lipoprotein), LDL (low density lipoprotein), cholesterol, triglycerides, HbA1c and blood cell counts (erythrocytes, leukocytes, neutrophil granulocytes, lymphocytes, monocytes, thrombocytes) were examined at the central laboratory of the Heidelberg university hospital. More advanced metabolic blood biomarkers beyond clinical routine were assessed at the Department of Epidemiology at the German Cancer Research Center (DKFZ), Heidelberg: i.e., TNF-alpha (tumor-necrose-factor-alpha), IL-6 (interleukin-6), IFN-gamma (interferon-gamma), adiponectin, leptin and CRP (C-reactive protein). Specifics about the blood parameter analyses have previously been published in detail (Schubel et al., 2018).

### MR-imaging

The MRI scans were conducted using a 1.5 T scanner (MAGNETOM Aera; Siemens Healthineers, Erlangen, Germany). The same MR-protocol, hardware and software were used throughout every MR scan at baseline, week 12 and week 50. The detailed MRI-protocol is published previously (Schubel et al., 2016).

**FIGURE 1:**
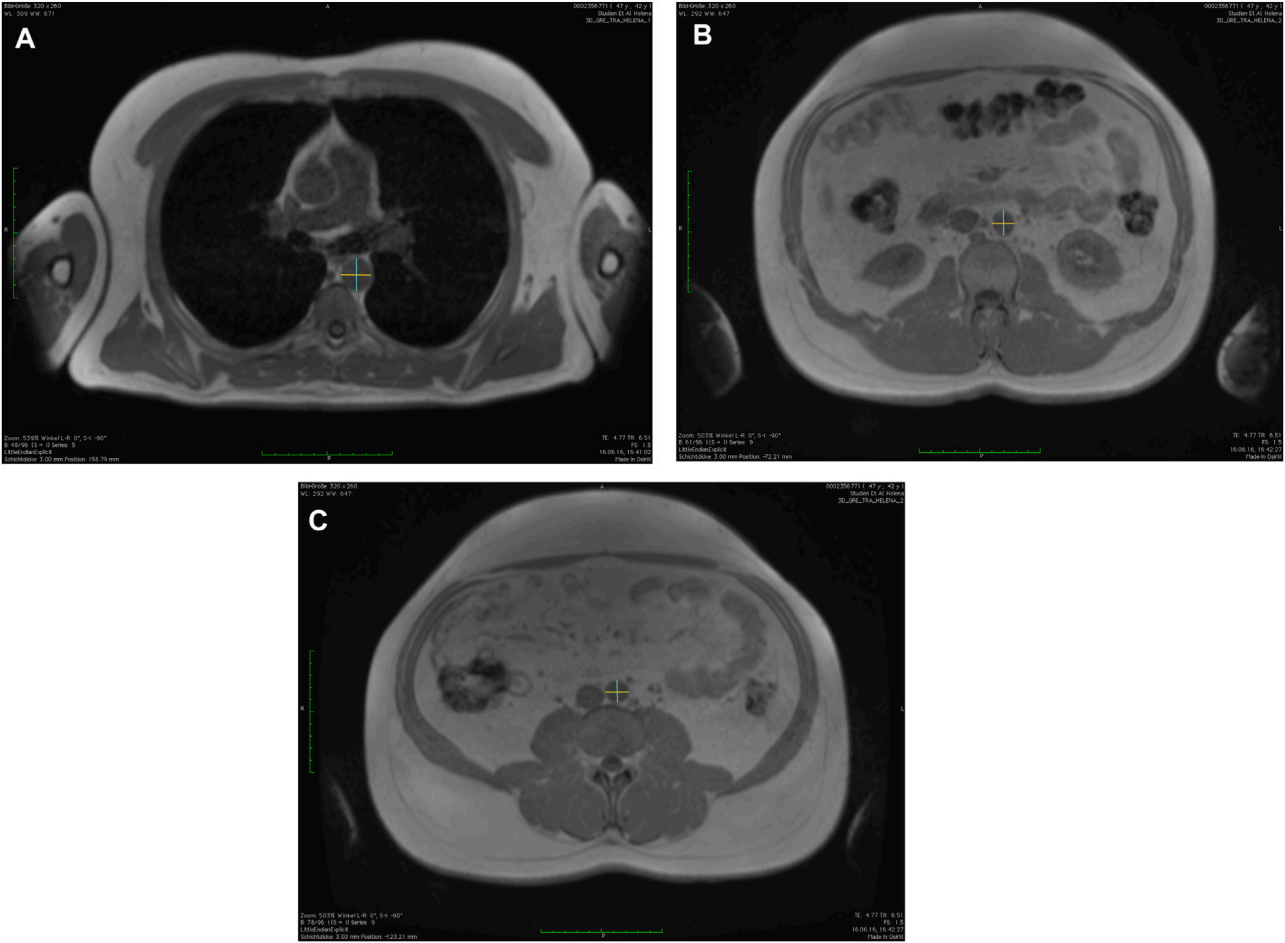
Measurements of the aortic diameters using the in-phase images of the T1-weighted-3D-VIBE two-point Dixon sequence with a post-processing software (OsiriX, Pixmeo SARL, Bernex, Switzerland) in anterior-posterior and left-right orientation at **(A)** descending thoracic aorta, **(B)** infrarenal abdominal aorta, and **(C)** directly above the aortic bifurcation.

The aortic diameters were manually measured using the in-phase of the T1-weighted-3D-VIBE two-point Dixon sequence at three anatomically defined locations in anterior-posterior and left-right orientation (calculation of mean value of anterior-posterior and left-right orientation) using a post-processing software (OsiriX, Pixmeo SARL, Bernex, Switzerland, see Figure 1) (Lehman et al., 2010; Rogers et al., 2013; Schubel et al., 2016). The locations were determined in accordance with the Framingham-Heart study (Lehman et al., 2010; Rogers et al., 2013; Qazi et al., 2017).

First, descending thoracic aorta (AD) at the level of the right pulmonary artery; secondly, the infrarenal abdominal aorta (INF; defined as 17 slides (5.1 cm) above the aortic bifurcation); and thirdly, directly above the aortic bifurcation (BIF) (Lehman et al., 2010; Rogers et al., 2013; Qazi et al., 2017). The measurements were done consecutively for each time point with the order of participants being randomized for each time point and each reader (S.S. and M.F.) separately by using a randomization tool (Excel, Microsoft, Redmont, United States).

Intra- and inter-reader agreement of the aortic diameter readings were assessed by calculating intraclass correlation coefficient (ICC) estimates and their 95% confident intervals using the psych package in R with a two-way mixed-effects, single measurement ICC model (Koo and Li, 2016). Moreover, smallest real differences (SRD) according to Bland and Altman were obtained as a measure of absolute reliability (Bland and Altman, 1986).

Reader 1 (SS) performed the baseline diameter readings twice for 40 participants for the estimation of intra-reader reliability. For the estimation of inter-reader agreement, both Reader 1 (SS) and Reader 2 (MF) performed the artic diameter readings for all participants at baseline (n = 144). The ICC analysis revealed an excellent intra-reader agreement for the readings of the baseline thoracic aorta diameter (0.97; 0.95–0.98, SRD: 0.11) and abdominal aorta diameter diameters (0.96; 0.93–0.98, SRD: 0.11). The intra-reader agreement for the baseline diameter of the aortic bifurcation readings was rated as good (0.93; 0.89–0.96, SRD: 0.13). With respect to inter-reader agreement, the readings of diameter of the descending thoracic aorta showed an excellent agreement 0.95 (0.94–0.96, SRD: 0.16), whereas the readings for both diameter of aortic bifurcation (0.83; 0.78–0.86, SRD: 0.20) and diameter of the infrarenal abdominal (0.87; 0.83–0.90, SRD: 0.22) showed good agreement.

The detailed information regarding the MRI-based measurements of the visceral (VAT) and subcutaneous adipose tissue (SAT) as well as the liver fat content (LFC) can be found in our previous publications (Schubel et al., 2016; Kuhn et al., 2018; Schubel et al., 2019).

### Statistical analyses

The primary endpoint of the HELENA trial was to assess the hypothesis that ICR is superior to CCR regarding adipose tissue gene expression, body composition and metabolic profile This, however, was not confirmed by our previously published manuscripts, which did not show any significant differences between ICR and CCR regarding primary, secondary, and exploratory endpoints (Schubel et al., 2018; Pannen et al., 2021).

For the present analyses, participants were re-categorized in a post-hoc manner into weight loss quartiles (WL quartiles) based on the degree of weight loss after the 12-weeks intervention period, regardless of the dietary regimen used to achieve weight loss. On average, the weight change among participants in WL quartile one was 0.0 ± 0.2% (n = 36), −3.2 ± 0.1% among those in WL quartile 2 (n = 36), −6.1 ± 0.2% among those in WL quartile 3 (n = 36) and -11.3 ± 0.6% among those in WL quartile 4 (n = 36). By using these equally large quartiles a larger statistical power could achieved than by using *a priori* determined cut-off points.

Linear mixed models adjusted for age and sex as fixed effects and modelling participant identifiers as random effects were used to examine the effect of weight loss on changes in the aortic diameters at week 12 and also at week 50. *p*-values for differences in outcome parameters according to weight loss were obtained modelling interactions between weight loss on the continuous scale and time (“time-by-treatment interaction”).

Furthermore, we analyzed the association of the aortic diameters with anthropometric measurements and metabolic parameters i.e. blood pressure, ALT, AST, GGT, HDL, total cholesterol, triglycerides, fasting glucose, HbA1c, insulin, CRP, IFN-γ, TNF-α, IL-6, IL-8 adiponectin and leptin at baseline, week 12 and week 50 using Spearman’s correlations adjusted for age and sex. All statistical analyses were performed in R (The R Foundation for Statistical Computing, Vienna, Austria; version 3.6.1′).

## Results

### Characteristics of study participants at baseline

The characteristics of our study participants according to weight loss quartiles are presented in Table 1. Participants were generally comparable across all weight loss quartiles at baseline with respect to anthropometric and other metabolic parameters. Likewise, the diameters of the descending thoracic aorta, aorta at bifurcation and infrarenal abdominal aorta were similar across the weight loss quartiles at baseline (Table 1). With regards to age, participants in the fourth quartile of weight loss were slightly younger compared to those in the first, second and third quartiles. Parts of the data shown in Table 1 have previously been reported in a related publication (Jiang et al., 2019).

**TABLE 1:** Baseline characteristics according to weight loss quartiles, n = 144^a^.

	Q1 (n = 36)	Q2 (n = 36)	Q3 (n = 36)	Q4 (n = 36)
Men, n (%)	16 (44.4%)	21 (58.3%)	17 (47.2%)	18 (50.0%)
Women, n (%)	20 (55.6%)	15 (41.7%)	19 (52.8%)	18 (50.0%)
Age, y	51.0 ± 6.3	51.2 ± 8.3	51.2 ± 7.8	47.4 ± 8.3
BMI, kg/m2	32.1 ± 4.1	31.1 ± 3.7	30.9 ± 3.4	31.5 ± 3.7
VAT, L	5.3 ± 2.2	5.0 ± 2.2	4.8 ± 2.0	4.7 ± 2.0
SAT, L	13.1 ± 4.6	11.2 ± 2.8	12.1 ± 3.9	12.9 ± 4.0
Liver fat, %	7.1 ± 4.4	8.8 ± 7.8	7.9 ± 6.5	7.4 ± 4.9
Diameter of descending thoracic aorta, cm	2.36 ± 0.27	2.38 ± 0.28	2.32 ± 0.24	2.32 ± 0.26
Diameter of aortic bifurcation, cm	1.70 ± 0.23	1.72 ± 0.17	1.74 ± 0.17	1.72 ± 0.22
Diameter of infrarenal abdominal aorta, cm	1.79 ± 0.22	1.82 ± 0.20	1.84 ± 0.17	1.80 ± 0.23
Systolic BP	139.6 ± 11	132.2 ± 14	136.6 ± 14.4	140 ± 21.9
Diastolic BP	90.1 ± 8.1	86 ± 8.1	87.3 ± 7.7	86.9 ± 9.8
Leptin, ng/ml	29.7 ± 25.1	20.4 ± 19.9	21.2 ± 15.1	29.4 ± 29
HOMA-IR	3.4 ± 1.9	2.9 ± 1.8	2.5 ± 1.2	2.6 ± 1.3
Insulin, mU/L	14.7 ± 7.8	12.6 ± 7.4	10.8 ± 5.1	11.2 ± 5.4
Glucose, mg/dL	93.4 ± 7.9	93.2 ± 6.8	94.8 ± 6.8	91.8 ± 8.0
IGF-1	114.6 ± 34.3	124.3 ± 34.1	111.3 ± 33.3	105.1 ± 27.0
HbA1c	5.4 ± 0.4	5.5 ± 0.3	5.5 ± 0.3	5.4 ± 0.3
Triglycerides, mg/dL	139.4 ± 64.9	136.1 ± 89.3	143.9 ± 93.2	108.3 ± 53.5
Cholesterol, mg/dL	211.4 ± 34.1	202.1 ± 35.9	214.4 ± 36.0	203.2 ± 34.5
LDL, mg/dL	129.5 ± 26.0	120.7 ± 25.0	128.8 ± 26.5	128.7 ± 29.5
HDL, mg/dL	54.0 ± 15.0	52.6 ± 14.3	56.8 ± 13.7	52.9 ± 14.9
ALT, U/L	25.1 ± 7.2	31.5 ± 14.1	26.9 ± 12.1	24.4 ± 9.7
AST, U/L	21.8 ± 4	25.8 ± 6.9	22.5 ± 4.0	22.3 ± 5.0
GGT, U/L	29.7 ± 13.9	26.2 ± 16.1	30.4 ± 19.8	24.3 ± 12
CRP, mg/pL	7.0 ± 8.6	4.3 ± 5.4	3.7 ± 2.8	3.8 ± 3.8
TNFα, ng/μL	4.2 ± 2.6	4.4 ± 2.8	4.9 ± 2.6	4.1 ± 2.5
IFNγ, ng/μL	16.6 ± 15.8	16.7 ± 24.8	17.2 ± 16.6	11.6 ± 8.9
IL6, ng/μL	2.0 ± 1.7	1.8 ± 3.4	1.3 ± 0.8	1.3 ± 1.1
IL8, ng/μL	10.7 ± 4.4	14.0 ± 23.0	10.0 ± 4.7	10.6 ± 5.2
Erythrocytes	4.8 ± 0.4	4.8 ± 0.5	4.9 ± 0.4	4.8 ± 0.4
Leukocytes	6.4 ± 1.5	6.2 ± 1.5	6.3 ± 1.3	6.3 ± 1.2
Neutrophils	58.8 ± 6.3	56.3 ± 8.0	57.6 ± 7.0	56.8 ± 9.2
Lymphocytes	29.7 ± 6.2	30.9 ± 6.3	30.4 ± 7.0	31.1 ± 8.5
Monocytes	6.0 ± 1.4	7.1 ± 1.9	6.5 ± 1.4	6.3 ± 1.5
Thrombocytes	239.8 ± 48.6	237.2 ± 49.3	242.9 ± 48.4	247.3 ± 61.6

a n = 144. Data are presented as mean ± standard deviation. Abbreviations: ALT, alanine transaminase; AST, aspartame transaminase; BMI, body mass index; CRP, C-reactive protein; GGT, gamma-glutamyl transpeptidase; HDL, high-density lipoprotein; HOMA-IR: homeostatic model assessment for insulin resistance; IFNγ, interferon gamma; IGF-1, insulin-like growth factor 1; IL-6, interleukin six; IL-8, interleukin 8; LDL, low-density lipoprotein; SAT, subcutaneous adipose tissue; TNFα, tumor necrosis factor-α; VAT, visceral adipose tissue.

### Effect of weight loss on aortic diameters, blood pressure and metabolic paramaters

The relative changes in weight after the 12-weeks controlled intervention were 0.0 ± 0.2%, −3.2 ± 0.1%, −6.1 ± 0.2%, and −11.3 ± 0.6 among participants in weight loss quartiles 1, 2, 3 and 4 respectively.

As shown in Table 2, there was an overall significant difference of the changes in the diameter of the thoracic aorta (*p* = 0.031) and abdominal aorta (*p* = 0.010) across WL quartiles after 12 weeks, with participants in the highest quartile of WL achieving the greatest reduction in the aforementioned aortic diameters (Table 2). The changes in the diameter of aortic bifurcation after 12 weeks were not significant overall across WL quartiles, even though there was a reduction in aortic bifurcation diameter among participants in the WL quartile 4 (−2.7 ± 1.3). During the follow-up period i.e. after 50 weeks, only the changes in the diameter of the thoracic aorta were significantly different overall across WL quartiles (*p* < 0.001), despite reductions in all aortic parameters across all WL quartiles. Similar to observations after 12 weeks, the reduction in the diameter of the thoracic aorta was greatest among participants in WL quartile 4.

**TABLE 2:** Relative changes in aortic diameters and blood pressure according to weight loss quartiles after 12 and 50 weeks^a^.

	WL quartile	Week 12	p*	Week 50	p*
Diameter of descending	Q1	0.0 ± 1.1	0.031	-0.1 ± 0.8	<0.001
thoracic aorta, %	Q2	−1.2 ± 1.1		−1.9 ± 0.9	
	Q3	0.4 ± 0.6		−1.1 ± 0.6	
	Q4	−2.5 ± 0.5		−4.8 ± 1.1	
Diameter of aortic bifurcation, %	Q1	0.8 ± 1.3	0.151	−0.8 ± 1.0	0.39
	Q2	0.0 ± 1.1		−1.3 ± 0.9	
	Q3	0.3 ± 1.2		−0.6 ± 1.1	
	Q4	−2.7 ± 1.3		−1.9 ± 1.5	
Diameter of infrarenal aorta, %; abdominal aorta, %	Q1	1.1 ± 1.1	0.010	−1.1 ± 0.8	0.80
	Q2	0.3 ± 0.8		−2.4 ± 0.9	
	Q3	0.0 ± 0.8		−2.4 ± 0.7	
	Q4	−2.2 ± 0.8		−1.8 ± 1.4	
Systolic BP, mmHg, %	Q1	−0.7 ± 1.1	<0.001	−1.3 ± 1.5	0.023
	Q2	-2.8 ± 1.3		−0.1 ± 1.3	
	Q3	−2.7 ± 0.9		−2.4 ± 1.3	
	Q4	−7.0 ± 1.7		−5.2 ± 1.5	
Diastolic BP, mmHg, %	Q1	0.5 ± 1.1	<0.0001	−1.7 ± 1.3	0.015
	Q2	−3.2 ± 1.1		−1.2 ± 1.4	
	Q3	−2.9 ± 0.9		−2.9 ± 1.1	
	Q4	−7.0 ± 1.5		−4.7 ± 1.5	

a *n* = 144. Data are presented as mean ± standard error. Relative changes were computed as loge relative changes, with baseline measurements as reference, i.e. log (week 12/baseline) * 100 for week 12 and log (week 50/baseline) * 100. *p*-values for time-by-weight change interactions were calculated using linear mixed models, adjusted for age and sex, and modelling weight loss (%) on the continuous scale. Abbreviations: BP, blood pressure.

Beyond the reductions in aortic parameters, weight loss was significantly associated with improvements in anthropometric measurements (BMI), blood pressure as well as other metabolic biomarkers, which have partly been published previously (see Supplementary Table S1) (Jiang et al., 2019).

With respect to blood pressure, systolic blood pressure (Systolic BP) decreased significantly across all WL quartiles, both after 12 (*p* < 0.001) and 50 weeks (*p* = 0.023, see Table 2). Similarly, weight loss was associated with reductions in diastolic blood pressure after 12 weeks (*p* < 0.0001) and also after 50 weeks (*p* = 0.015, see Table 2).

### Association of aortic diameters with anthropometric and metabolic markers

**FIGURE 2:**
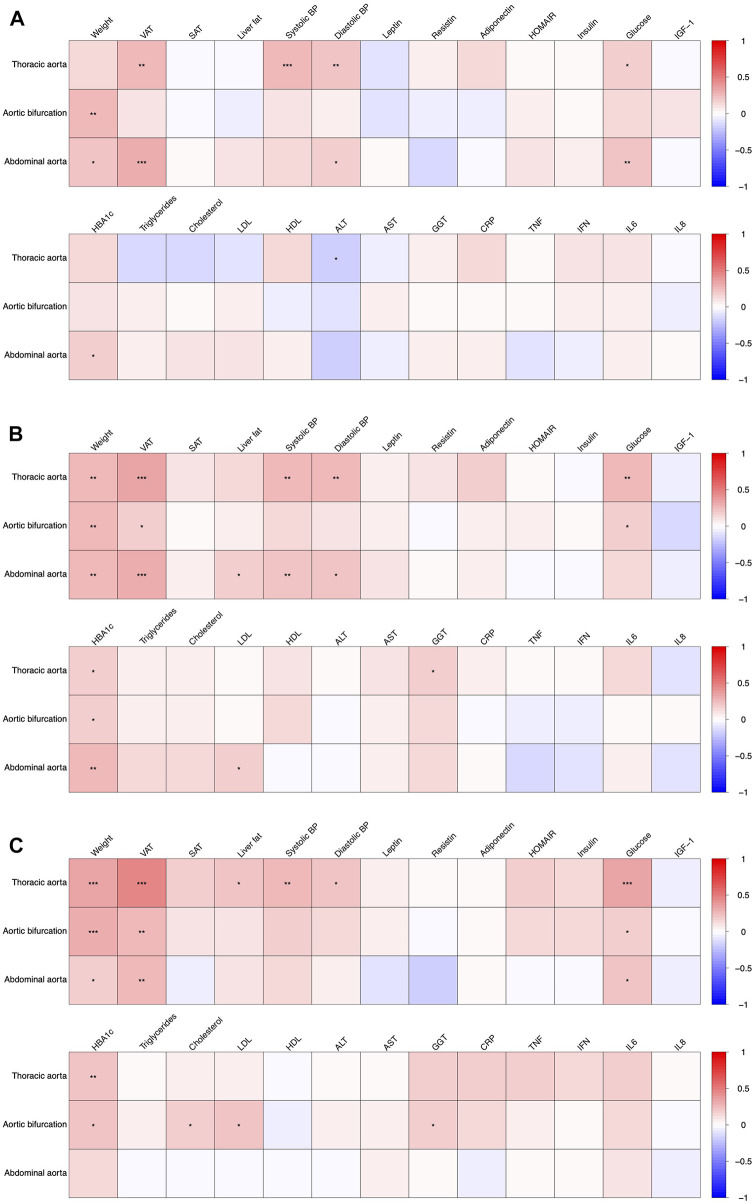
Association of aortic diameters with anthropometric and metabolic parameters at baseline (**(A)**, T0), after 12 weeks of intervention (**(B)**, T1) and after 50 weeks (**(C)**, T3). Significant associations are indicated with asterisks (*), (**) and (***) denoting a *p* < 0.05, *p* < 0.01 and *p* < 0.001, respectively.

The association of the aortic diameters with metabolic parameters at baseline, at weeks 12 and 50 are depicted in Figure 2. For further detail, the associations at baseline, weeks 12 and 50 are shown in Supplementary Tables S2-S4, respectively.

The diameter of the aortic bifurcation and infrarenal abdominal aorta showed a weak positive association with body weight at baseline, rho = 0.24 and 0.20 respectively (Figure 2, see also Supplementary Table S2). These associations were consistent at weeks 12 and 50 (Figure 2, Supplementary Tables S3, S4). There was also a weak positive association of VAT with the diameters of both the descending thoracic aorta and the infrarenal abdominal aorta at baseline, rho = 0.26 and 0.29 respectively. More pronounced weak-to-modest positive associations were observed at weeks 12 and 50 (rho = 0.36 and 0.32; and rho = 0.47 and 0.26, respectively, see Supplementary Tables S3, S4, Figure 2). The diameters of the descending thoracic aorta showed a weak-to-modest positive association with both systolic and diastolic blood pressure as well as plasma glucose concentrations across all study timepoints i.e. baseline (Figure 2), week 12 and 50 (Figure 2, Supplementary Tables S3, S4). The diameters of the infrarenal abdominal aorta depicted a weak-to-modest positive association with diastolic blood pressure, plasma glucose concentrations and HbA1c. No further consistent associations were observed.

## Discussion

In the present study, we investigated the effect of a moderate weight loss intervention on the diameter of the aorta measured at the descending thoracic aorta, aortic bifurcation and infrarenal abdominal aorta as proxy for the risk of aortic dilatation and aortic aneurysm in overweight or obese non-smoking, but otherwise healthy participants of the HELENA Trial. Furthermore, we assessed the associations of the aortic diameter with body fat depots, anthropometrics, blood pressure as well as markers of metabolism.

The main finding of our study is a significant decrease in diameter of the descending thoracic and infrarenal abdominal aorta after 12 weeks, with the greatest diameter reductions in participants with the highest weight loss after intervention. Albeit not statistically significant across the four weight loss quartiles, the diameter of the aortic bifurcation decreased also among individuals in the highest weight loss quartile after 12 weeks. After 50 weeks, only the reductions of diameter of the descending thoracic aorta were statistically still significant across the weight loss quartiles, despite non-significant reductions of the infrarenal abdominal aorta and aortic bifurcation. Again, reductions of aortic diameters were more pronounced in the highest weight loss quartile.

Thus, our results indicate a reduction of the aortic diameter especially in the region of the descending thoracic and infrarenal abdominal aorta after 12 weeks of weight loss intervention. These findings were more pronounced in participants achieving a higher weight loss after the caloric restriction intervention (i.e. >7.5% of weight loss in the highest quartile). Furthermore, the reductions of aortic diameter were still present after the follow-up time after 50 weeks. To the best of our knowledge, so far no comparable studies in humans examining the change of aortic diameter after weight loss using cross sectional imaging techniques have been carried out. However, there exist several studies analyzing the effect of weight loss on the arterial stiffness as marker for aortic function, which plays an important role in the pathophysiology of aortic dilatation and cardiovascular diseases. Ikonomidis et al. could show an improved aortic function in 60 individuals after bariatric surgery (Ikonomidis et al., 2007). Similarly, Gul et al. observed an improvement of aortic stiffness 1 year after laparoscopic sleeve gastrectomy in 53 obese individuals (Gul et al., 2021). Also, Brinkley et al. could demonstrate an improved aortic stiffness in elderly obese after moderate and intensive caloric restriction combined with aerobic exercise training, but not with aerobic exercise training alone (Brinkley et al., 2021).

In an experimental study with mice by Liu et al., a 12 weeks caloric restriction prevented the development of angiotensin II-induced abdominal aortic aneurysm, which, according to the authors, seemed to be based on an upregulation of Sirtuin1 in vascular smooth muscle cells (Liu et al., 2016).

Beyond weight loss, there are hints of the influence of diet on the development of an aortic aneurysm. Exemplarily, in 13.496 individuals of the ARIC (Atherosclerosis Risk in Communities) study, a diet according to the Dietary Approaches To Stop Hypertension‐style (DASH diet) and a higher consumption of fruit, vegetable, whole grain, nuts, legumes and low-fat dairy were associated with a lower risk for aortic aneurysm (Haring et al., 2018). Also in the prospective Malmö Diet and Cancer study with 26.000 individuals, a high intake of fruit, berries, vegetable, especially leaf vegetable, were associated with a decreased risk for the development of an aortic aneurysm (Bergwall et al., 2020).

With respect to blood pressure, systolic blood pressure (Systolic BP) decreased significantly across all WL quartiles, both after 12 (*p* < 0.001) and 50 weeks (*p* = 0.023). Similarly, weight loss was associated with reductions in diastolic blood pressure after 12 weeks (*p* < 0.0001) and also after 50 weeks (*p* = 0.015). The influence of weight reductions on blood pressure is well-established and exemplary described in a meta-analysis of randomized controlled trials by Neter et al. observing greater reductions of blood pressure with increasing amounts of weight loss (Neter et al., 2003). Our correlations between BP and AD are also in line with results from the Framingham trial (Rogers et al., 2013).

Our data suggest that such reductions in blood pressure may be in part due to a decrease in the diameter of the thoracic aorta, given that both parameters may be causally associated according to a recent Mendelian randomization study (John DePaolo et al., 2021). As aortic diameters are thought to be age-dependent (Rogers et al., 2013), weight loss may reverse an aspect of vascular ageing. However, our finding requires further experimental follow-up studies to better understand potential underlying mechanisms, and to assess whether weight loss, aortic diameters and aneurysms may be causally linked’

In our study, we further assessed the associations of the aortic diameters with body fat depots, anthropometrics, blood pressure as well as markers of metabolism. We showed positive associations of the aortic diameters with both weight and VAT consistently over the three time points. Regarding weight, a comparable positive association with aortic diameters was also seen in others studies, albeit they used the BMI as measure (Lederle et al., 2000; Allison et al., 2008; Kent et al., 2010). Regarding VAT, Gorter et al. could show that VAT accumulation is associated with larger aortic diameters in patients with arterial disease (Gorter et al., 2008). Also a study by Jiang et al. showed a positive association of visceral fat with a dilatation of the proximal aorta (Jiang et al., 2018). Thanassoulis et al. could demonstrate in a community-based sample of 3,000 individuals of the Framingham Trial, that CT-measured VAT and periaortic fat were positively associated with thoracic and abdominal aortic dimensions (Thanassoulis et al., 2012).

Regarding the other fat depots, LFC was weakly associated with the aortic diameters after week 12 and 50 in our study, but not at baseline (see Supplementary Tables S2, S3 and Figure 2), while SAT showed no significant associations with the aortic diameters. In a cross-sectional investigation embedded in two independent population-based studies (KORA and SHIP study), Cai et al. could also demonstrate a positive association with increased liver fat content and aortic diameters (ascending, descending and infrarenal aorta), which however attenuated after correcting with other cardiovascular risk factors including BMI (Cai et al., 2021). With respect to SAT, aortic dilatation also showed no association with SAT in a study by Apolini et al. (Apoloni et al., 2019).

Our study is the first study to examine the impact of a weight loss intervention on the aortic diameter over three time points with a follow-up of 1 year in a large cohort of obese study participants. Moreover, our study has the strength to use not only aortic diameters measured at MR-images, but also has the availability of MRI-based body fat depots like VAT, SAT and LFC, blood pressure, anthropometrics as well as a wide spectrum of metabolic biomarkers each measured at three time points.

This study is subject to following limitations: we only included healthy obese and overweight individuals and thus have no unconfined insights into patients with chronic diseases like diabetes. Also, we did not include patients with aortic aneurysm or other aortic diseases as the impact of weight loss as therapy for aortic aneurysm was not part of this study. The study was not designed to assess incident aortic aneurysms either, and aortic diameters were only used as proxy for increased risk of aortic aneurysms. Thus, future studies designed to assess aortic aneurysm in relation to obesity and weight loss are needed before stronger conclusions on the preventive potential of calorie restriction can be drawn (Eckstein and Maegdefessel, 2020). Additionally, only non-smokers were included in the HELENA Trial. Therefore, the results are not fully applicable to smokers, who are at higher risk for developing aortic aneurysms. The MR images were non-ECG-gated; thus, we were unable to adequately measure the diameters at the ascending aorta because of motion artifacts. Finally, while the present study serves as an excellent model to investigate the effects of intentional dietary weight loss on aortic diameters, our analyses were exploratory, i.e. not pre-defined in the initial trial protocol. Therefore, we cannot proof that simultaneous changes in blood pressure and aortic diameters upon weight loss in our study are causally linked.

## Conclusion

In conclusion, a moderate, dietary induced weight loss in overweight and obese non-smokers is associated with a significant decrease of aortic diameters as assessed by follow-up MRI. Furthermore, aortic diameters were positively associated with weight, VAT, glucose, HbA1c and with both systolic and diastolic blood pressure. Thus, weight loss induced by calorie restriction may serve to prevent aortic dilatation and thus the development of aortic aneurysms in obese and overweight individuals. Further studies are needed to investigate the impact of weight loss on aortic diameters in obese individuals with chronic diseases such as diabetes or risk factors such as smoking.

## Data Availability

The raw data supporting the conclusions of this article will be made available by the authors, without undue reservation.
