# Supplementary material for: Changes in aortic diameter induced by weight loss: The HELENA trial- whole-body MR imaging in a dietary intervention trial
Source: Front Physiol. 2022 Sep 20;13:976949. doi: 10.3389/fphys.2022.976949 (PMC9531129; doi:10.3389/fphys.2022.976949)
Supplement: Supplementary file 1 [file DataSheet1.docx]

Supplementary Material

**Supplement table 1.** Relative changes in metabolic parameters according to weight loss quartiles after 12 and 50 weeks

|  | WL quartile | Week 12 | p | Week 50 | p |
| --- | --- | --- | --- | --- | --- |
| BMI, kg/m^2^, % | Q1 | 0.0 ± 0.2 | <0.0001 | 1.2 ± 0.5 | <0.0001 |
|  | Q2 | -3.2 ± 0.1 |  | -1.3 ± 0.5 |  |
|  | Q3 | -6.1 ± 0.2 |  | -4.3 ± 0.7 |  |
|  | Q4 | -11.3 ± 0.6 |  | -11.1 ± 1.5 |  |
| VAT, L, % | Q1 | -0.1 ± 0.9 | <0.0001 | 4.9 ± 2.0 | <0.0001 |
|  | Q2 | -10.0 ± 1.3 |  | -7.1 ± 2.5 |  |
|  | Q3 | -18.1 ± 1.3 |  | -16.7 ± 2.6 |  |
|  | Q4 | -33.9 ± 3.4 |  | -45.4 ± 7.3 |  |
| SAT, L, % | Q1 | -0.6 ± 1.3 | <0.0001 | 3.9 ± 1.7 | <0.0001 |
|  | Q2 | -7.1 ± 1.0 |  | -4.3 ± 1.5 |  |
|  | Q3 | -13.0 ± 1.1 |  | -9.1 ± 2.4 |  |
|  | Q4 | -27.0 ± 2.7 |  | -26.4 ± 5.3 |  |
| Liver fat, % | Q1 | -2.6 ± 5.4 | <0.0001 | -0.3 ± 6.1 | <0.0001 |
|  | Q2 | -25.9 ± 5.7 |  | -12.3 ± 4.8 |  |
|  | Q3 | -35.8 ± 5.5 |  | -35.9 ± 7.3 |  |
|  | Q4 | -63.9 ± 10.3 |  | -56.9 ± 10.5 |  |
| Leptin, ng/ml, % | Q1 | -17 ± 9.3 | 0.006 | -3.2 ± 9 | 0.002 |
|  | Q2 | -37.5 ± 6.9 |  | -20.6 ± 8.2 |  |
|  | Q3 | -50.5 ± 6.1 |  | -38.8 ± 10.2 |  |
|  | Q4 | -81.7 ± 10.8 |  | -67.3 ± 13.5 |  |
| HOMA-IR, % | Q1 | -7.9 ± 7.6 | 0.85 | -20.6 ± 9.0 | 0.42 |
|  | Q2 | -12.4 ± 7.7 |  | -16.0 ± 6.2 |  |
|  | Q3 | -24.3 ± 7.3 |  | -38.5 ± 8.4 |  |
|  | Q4 | -21.4 ± 7.6 |  | -45.1 ± 9.7 |  |
| Insulin, mU/L, % | Q1 | -5.1 ± 7.1 | 0.63 | -16.9 ± 8.2 | 0.42 |
|  | Q2 | -7.3 ± 7.0 |  | -13.7 ± 5.2 |  |
|  | Q3 | -16.0 ± 6.4 |  | -31.0 ± 7.7 |  |
|  | Q4 | -17.0 ± 6.9 |  | -38.8 ± 8.8 |  |
| Glucose, mg/dL, % | Q1 | -2.8 ± 1.3 | 0.14 | -3.8 ± 1.4 | 0.08 |
|  | Q2 | -5.0 ± 1.6 |  | -2.4 ± 1.4 |  |
|  | Q3 | -8.3 ± 1.6 |  | -7.5 ± 1.4 |  |
|  | Q4 | -4.6 ± 1.3 |  | -6.6 ± 1.5 |  |
| IGF-1, % | Q1 | 2.6 ± 4.2 | 0.002 | 3.4 ± 5.4 | 0.098 |
|  | Q2 | -6.1 ± 4.5 |  | -7.6 ± 6.6 |  |
|  | Q3 | 1.6 ± 6.3 |  | -1.1 ± 4.5 |  |
|  | Q4 | 24.3 ± 5.7 |  | 14.6 ± 5.4 |  |
| HbA1c, % | Q1 | -0.4 ± 0.5 | 0.36 | -0.3 ± 0.5 | <0.0001 |
|  | Q2 | 0.4 ± 0.6 |  | -1.5 ± 0.8 |  |
|  | Q3 | -0.7 ± 0.6 |  | -1.7 ± 0.6 |  |
|  | Q4 | -1.5 ± 0.6 |  | -4.2 ± 0.8 |  |
| Triglycerides, mg/dL, % | Q1 | -4.2 ± 5.6 | 0.055 | -10.0 ± 6.0 | 0.20 |
|  | Q2 | -17.2 ± 4.6 |  | -9.8 ± 5.1 |  |
|  | Q3 | -25.6 ± 5.0 |  | -26.1 ± 7.2 |  |
|  | Q4 | -22.9 ± 6.7 |  | -20.4 ± 7.5 |  |
| Cholesterol, mg/dL, % | Q1 | -5.2 ± 1.5 | <0.0001 | -0.1 ± 1.8 | 0.003 |
|  | Q2 | -8.8 ± 2.4 |  | -1.7 ± 1.9 |  |
|  | Q3 | -11.2 ± 2.0 |  | -5.1 ± 2.2 |  |
|  | Q4 | -16.3 ± 2.1 |  | -7.1 ± 2.7 |  |
| LDL, mg/dL, % | Q1 | -4.3 ± 2.4 | <0.001 | 4.1 ± 2.7 | 0.001 |
|  | Q2 | -4.2 ± 2.7 |  | 1.4 ± 2.9 |  |
|  | Q3 | -8.0 ± 2.7 |  | -3.0 ± 3.1 |  |
|  | Q4 | -15.2 ± 2.8 |  | -6.3 ± 3.7 |  |
| HDL, mg/dL, % | Q1 | -6.4 ± 2.3 | 0.056 | -3.5 ± 2.6 | 0.34 |
|  | Q2 | -8.0 ± 2.2 |  | -2.0 ± 2.5 |  |
|  | Q3 | -12.2 ± 2.4 |  | -1.6 ± 2.2 |  |
|  | Q4 | -13.2 ± 2.6 |  | -2.0 ± 2.3 |  |
| ALT, U/L, % | Q1 | -10.0 ± 5.2 | 0.003 | -3.2 ± 5.9 | 0.024 |
|  | Q2 | -16.0 ± 4.8 |  | -12.0 ± 5.3 |  |
|  | Q3 | -18.1 ± 5.7 |  | -19.9 ± 5.9 |  |
|  | Q4 | -32.3 ± 4.7 |  | -22.5 ± 8.1 |  |
| AST, U/L, % | Q1 | -7.8 ± 3.4 | 0.11 | -7.4 ± 3.8 | 0.29 |
|  | Q2 | -12.1 ± 4.1 |  | -15.8 ± 4.0 |  |
|  | Q3 | -8.2 ± 4.5 |  | -14.6 ± 4.5 |  |
|  | Q4 | -16.7 ± 3.3 |  | -14.7 ± 4.4 |  |
| GGT, U/L, % | Q1 | -11.3 ± 3.8 | <0.0001 | -3.3 ± 4.8 | 0.004 |
|  | Q2 | -12.4 ± 3.2 |  | -5.6 ± 6.5 |  |
|  | Q3 | -31.7 ± 4.6 |  | -23.0 ± 5.2 |  |
|  | Q4 | -47.5 ± 6.1 |  | -36.1 ± 7.5 |  |
| CRP, mg/pL, % | Q1 | -17.6 ± 13.9 | 0.74 | 7.0 ± 19.4 | 0.14 |
|  | Q2 | -12.8 ± 13.2 |  | -17.5 ± 14.8 |  |
|  | Q3 | -22.2 ± 13.1 |  | -38.7 ± 11.9 |  |
|  | Q4 | -36.0 ± 11.4 |  | -48.2 ± 14.2 |  |
| TNFα, ng/μL, % | Q1 | 0.9 ± 2.7 | 0.77 | 99.1 ± 11.4 | 0.76 |
|  | Q2 | 0.1 ± 3.3 |  | 90.8 ± 9.9 |  |
|  | Q3 | 1.4 ± 2.6 |  | 75.8 ± 12.0 |  |
|  | Q4 | -4.1 ± 3.4 |  | 95.8 ± 11.3 |  |
| IFNγ, ng/μL, % | Q1 | 5.8 ± 10.9 | 0.77 | 99.6 ± 15.7 | 0.44 |
|  | Q2 | 2.7 ± 16.9 |  | 114.5 ± 17.5 |  |
|  | Q3 | 0.5 ± 11.5 |  | 74.0 ± 13.9 |  |
|  | Q4 | 1.0 ± 14.7 |  | 110.0 ± 23.0 |  |
| IL6, ng/μL, % | Q1 | -1.0 ± 10.2 | 0.48 | 138.0 ± 12.7 | 0.094 |
|  | Q2 | 3.7 ± 10.1 |  | 137.4 ± 11.8 |  |
|  | Q3 | 11.7 ± 8.8 |  | 121.1 ± 13.5 |  |
|  | Q4 | -2.7 ± 9.1 |  | 129.8 ± 13.8 |  |
| IL8, ng/μL, % | Q1 | -11.3 ± 5.8 | 0.51 | 21.5 ± 7.2 | 0.69 |
|  | Q2 | 10.2 ± 5.3 |  | 28.2 ± 9.7 |  |
|  | Q3 | -3.6 ± 5.0 |  | 37.1 ± 6.6 |  |
|  | Q4 | 2.0 ± 4.7 |  | 21.7 ± 8.8 |  |
| Erythrocytes, % | Q1 | -3.0 ± 0.8 | 0.003 | -2.8 ± 0.8 | 0.012 |
|  | Q2 | -1.4 ± 0.7 |  | -2.3 ± 0.7 |  |
|  | Q3 | -2.5 ± 0.8 |  | -2.7 ± 0.9 |  |
|  | Q4 | -4.7 ± 0.9 |  | -4.7 ± 1.0 |  |
| Leukocytes, % | Q1 | -4.0 ± 2.9 | 0.079 | 0.5 ± 3.3 | 0.022 |
|  | Q2 | -6.7 ± 2.5 |  | -8.4 ± 2.3 |  |
|  | Q3 | -4.1 ± 3.0 |  | -9.9 ± 2.6 |  |
|  | Q4 | -10.4 ± 2.4 |  | -9.3 ± 3.5 |  |
| Neutrophils, % | Q1 | -3.3 ± 1.6 | 0.012 | -2.9 ± 1.6 | 0.072 |
|  | Q2 | -1.4 ± 1.7 |  | 0.9 ± 1.8 |  |
|  | Q3 | 2.1 ± 1.5 |  | -1.3 ± 1.3 |  |
|  | Q4 | 2.8 ± 1.5 |  | 3.6 ± 2.0 |  |
| Lymphocytes, % | Q1 | 1.3 ± 2.7 | 0.029 | 2.6 ± 2.8 | 0.13 |
|  | Q2 | 0.3 ± 3.2 |  | -1.0 ± 3.2 |  |
|  | Q3 | -6.6 ± 3.2 |  | 0.9 ± 2.2 |  |
|  | Q4 | -6.3 ± 3.2 |  | -5.1 ± 3.7 |  |
| Monocytes, % | Q1 | 10.0 ± 2.8 | 0.88 | 4.8 ± 3.4 | 0.30 |
|  | Q2 | 1.2 ± 3.3 |  | -4.1 ± 3.0 |  |
|  | Q3 | 4.1 ± 3.4 |  | -2.0 ± 3.2 |  |
|  | Q4 | 5.7 ± 3.3 |  | -3.1 ± 3.6 |  |
| Thrombocytes, % | Q1 | -2.6 ± 1.5 | <0.0001 | 2.7 ± 1.5 | <0.001 |
|  | Q2 | -7.9 ± 1.9 |  | -2.3 ± 2.1 |  |
|  | Q3 | -6.1 ± 1.7 |  | -1.2 ± 2.0 |  |
|  | Q4 | -13.7 ± 2.0 |  | -6.4 ± 2.5 |  |

^1^n= 144. Data are presented as mean ± standard error. Relative changes were computed as loge relative changes, with baseline measurements as reference, i.e. log (week 12/baseline) * 100 for week 12 and log (week 50/baseline) * 100. p-values for time-by-weight change interactions were calculated using linear mixed models, adjusted for age and sex, and modelling weight loss (%) on the continuous scale. Abbreviations: ALT, alanine transaminase; AST, aspartame transaminase; BMI, body mass index; CRP, C-reactive protein; GGT, gamma-glutamyl transpeptidase; HDL, high-density lipoprotein; HOMA-IR: homeostatic model assessment for insulin resistance; IFNγ, interferon gamma; IGF-1, insulin-like growth factor 1; IL-6, interleukin 6; IL-8, interleukin 8; LDL, low-density lipo-protein; SAT, subcutaneous adipose tissue; TNFα, tumor necrosis factor-α; VAT, visceral adipose tissue

**Supplement table 2**: Spearman’s correlations of the aortic diameters with anthropometric measurements, fat depots, blood pressure and metabolic parameters at baseline adjusted for age and sex, n=144

|  | **Descending thoracic**  **aorta** | | **Aortic bifurcation** | | **Infrarenal abdominal aorta** | |
| --- | --- | --- | --- | --- | --- | --- |
|  | **r** | **p** | **r** | **p** | **r** | **p** |
| **Weight** | 0.15 | 0.07 | 0.24 | **<0.01** | 0.20 | **0.02** |
| **VAT** | 0.26 | **<0.01** | 0.09 | 0.28 | 0.29 | **<0.001** |
| **SAT** | -0.01 | 0.91 | -0.01 | 0.91 | 0.01 | 0.94 |
| **Liver fat** | -0.01 | 0.92 | -0.06 | 0.45 | 0.09 | 0.27 |
| **Systolic BP** | 0.28 | **<0.001** | 0.08 | 0.31 | 0.16 | 0.06 |
| **Diastolic BP** | 0.22 | **0.01** | 0.08 | 0.36 | 0.19 | **0.02** |
| **Leptin** | -0.12 | 0.16 | -0.09 | 0.26 | 0.03 | 0.72 |
| **Resistin** | 0.04 | 0.60 | -0.05 | 0.55 | -0.14 | 0.09 |
| **Adiponectin** | 0.13 | 0.12 | -0.07 | 0.40 | -0.01 | 0.91 |
| **HOMAIR** | 0.02 | 0.86 | 0.04 | 0.60 | 0.08 | 0.33 |
| **Insulin** | 0.00 | 1.00 | 0.02 | 0.79 | 0.07 | 0.44 |
| **Glucose** | 0.17 | **0.04** | 0.15 | 0.08 | 0.24 | <**0.01** |
| **IGF-1** | -0.03 | 0.74 | 0.08 | 0.33 | -0.02 | 0.81 |
| **HbA1c** | 0.15 | 0.07 | 0.08 | 0.34 | 0.17 | **0.05** |
| **Triglycerides** | -0.14 | 0.08 | 0.04 | 0.60 | 0.07 | 0.41 |
| **Cholesterol** | -0.12 | 0.14 | 0.02 | 0.79 | 0.09 | 0.31 |
| **LDL** | -0.10 | 0.24 | 0.05 | 0.56 | 0.10 | 0.24 |
| **HDL** | 0.13 | 0.12 | -0.05 | 0.56 | 0.06 | 0.45 |
| **ALT** | -0.20 | **0.02** | -0.09 | 0.27 | -0.16 | 0.05 |
| **AST** | -0.05 | 0.53 | 0.06 | 0.47 | -0.05 | 0.52 |
| **GGT** | 0.06 | 0.50 | 0.04 | 0.68 | 0.08 | 0.37 |
| **CRP** | 0.15 | 0.07 | 0.01 | 0.91 | 0.05 | 0.54 |
| **TNF** | 0.03 | 0.69 | 0.04 | 0.66 | -0.11 | 0.21 |
| **IFN** | 0.11 | 0.17 | 0.04 | 0.63 | -0.06 | 0.45 |
| **IL6** | 0.11 | 0.21 | 0.05 | 0.54 | 0.05 | 0.58 |
| **IL8** | -0.01 | 0.94 | -0.06 | 0.48 | 0.03 | 0.71 |

Abbreviations: ALT, alanine transaminase; AST, aspartame transaminase; BMI, body mass index; CRP, C-reactive protein; GGT, gamma-glutamyl transpeptidase; HDL, high-density lipoprotein; HOMA-IR: homeostatic model assessment for insulin resistance; IFNγ, interferon gamma; IGF-1, insulin-like growth factor 1; IL-6, interleukin 6; IL-8, interleukin 8; LDL, low-density lipoprotein; SAT, subcutaneous adipose tissue; TNFα, tumor necrosis factor-α; VAT, visceral adipose tissue

**Supplement table 3**: Spearman’s correlations of the aortic diameters with anthropometric measurements, fat depots, blood pressure and metabolic parameters at week 12 adjusted for age and sex, n=144

|  | **Descending thoracic**  **aorta** | | **Aortic bifurcation** | | **Infrarenal abdominal aorta** | |
| --- | --- | --- | --- | --- | --- | --- |
|  | **r** | **p** | **r** | **p** | **r** | **p** |
| **Weight** | 0.24 | **<0.01** | 0.27 | **<0.01** | 0.26 | **<0.01** |
| **VAT** | 0.36 | **<0.0001** | 0.19 | **0.03** | 0.32 | **<0.001** |
| **SAT** | 0.10 | 0.25 | 0.03 | 0.70 | 0.08 | 0.36 |
| **Liver fat** | 0.13 | 0.13 | 0.04 | 0.64 | 0.19 | **0.03** |
| **Systolic BP** | 0.25 | **<0.01** | 0.14 | 0.10 | 0.22 | **0.01** |
| **Diastolic BP** | 0.25 | **<0.01** | 0.09 | 0.30 | 0.21 | **0.01** |
| **Leptin** | 0.06 | 0.47 | 0.08 | 0.39 | 0.10 | 0.26 |
| **Resistin** | 0.09 | 0.31 | -0.03 | 0.69 | 0.03 | 0.74 |
| **Adiponectin** | 0.16 | 0.06 | 0.06 | 0.50 | 0.07 | 0.44 |
| **HOMAIR** | 0.02 | 0.83 | 0.04 | 0.63 | -0.01 | 0.89 |
| **Insulin** | -0.02 | 0.83 | 0.02 | 0.79 | -0.03 | 0.73 |
| **Glucose** | 0.25 | **<0.01** | 0.20 | **0.02** | 0.14 | 0.12 |
| **IGF-1** | -0.05 | 0.56 | -0.13 | 0.12 | -0.08 | 0.39 |
| **HbA1c** | 0.20 | **0.02** | 0.18 | **0.04** | 0.25 | **<0.01** |
| **Triglycerides** | 0.07 | 0.41 | 0.06 | 0.51 | 0.12 | 0.15 |
| **Cholesterol** | 0.05 | 0.55 | 0.08 | 0.38 | 0.15 | 0.07 |
| **LDL** | 0.01 | 0.87 | 0.03 | 0.74 | 0.17 | **0.05** |
| **HDL** | 0.10 | 0.25 | 0.13 | 0.13 | -0.03 | 0.77 |
| **ALT** | 0.01 | 0.86 | -0.02 | 0.81 | -0.03 | 0.69 |
| **AST** | 0.11 | 0.20 | 0.04 | 0.62 | 0.06 | 0.49 |
| **GGT** | 0.19 | **0.03** | 0.12 | 0.16 | 0.12 | 0.16 |
| **CRP** | 0.07 | 0.42 | -0.03 | 0.70 | 0.04 | 0.68 |
| **TNF** | 0.00 | 0.96 | -0.08 | 0.36 | -0.13 | 0.13 |
| **IFN** | 0.01 | 0.87 | -0.07 | 0.39 | -0.08 | 0.34 |
| **IL6** | 0.13 | 0.12 | 0.03 | 0.72 | 0.07 | 0.45 |
| **IL8** | -0.10 | 0.26 | 0.02 | 0.86 | -0.09 | 0.29 |

Abbreviations: ALT, alanine transaminase; AST, aspartame transaminase; BMI, body mass index; CRP, C-reactive protein; GGT, gamma-glutamyl transpeptidase; HDL, high-density lipoprotein; HOMA-IR: homeostatic model assessment for insulin resistance; IFNγ, interferon gamma; IGF-1, insulin-like growth factor 1; IL-6, interleukin 6; IL-8, interleukin 8; LDL, low-density lipoprotein; SAT, subcutaneous adipose tissue; TNFα, tumor necrosis factor-α; VAT, visceral adipose tissue

**Supplement table 4**: Spearman’s correlations of the aortic diameters with anthropometric measurements, fat depots, blood pressure and metabolic parameters at week 50 adjusted for age and sex, n=144

|  | **Descending thoracic**  **aorta** | | **Aortic bifurcation** | | **Infrarenal abdominal aorta** | |
| --- | --- | --- | --- | --- | --- | --- |
|  | **r** | **p** | **r** | **p** | **r** | **p** |
| **Weight** | 0.33 | **<0.001** | 0.30 | **<0.001** | 0.18 | **0.04** |
| **VAT** | 0.47 | **<0.0001** | 0.25 | **<0.01** | 0.26 | **<0.01** |
| **SAT** | 0.17 | 0.05 | 0.11 | 0.22 | -0.04 | 0.61 |
| **Liver fat** | 0.22 | **0.01** | 0.12 | 0.18 | 0.09 | 0.33 |
| **Systolic BP** | 0.26 | **<0.01** | 0.17 | 0.06 | 0.13 | 0.15 |
| **Diastolic BP** | 0.21 | **0.02** | 0.14 | 0.10 | 0.06 | 0.49 |
| **Leptin** | 0.06 | 0.53 | 0.05 | 0.59 | -0.10 | 0.28 |
| **Resistin** | 0.04 | 0.66 | -0.04 | 0.70 | -0.17 | 0.05 |
| **Adiponectin*** | - | - | - | - | - | - |
| **HOMAIR** | 0.16 | 0.07 | 0.14 | 0.11 | 0.00 | 0.97 |
| **Insulin** | 0.14 | 0.12 | 0.13 | 0.13 | -0.03 | 0.76 |
| **Glucose** | 0.34 | **<0.001** | 0.20 | **0.03** | 0.22 | **0.01** |
| **IGF-1** | -0.05 | 0.61 | -0.02 | 0.80 | -0.06 | 0.49 |
| **HbA1c** | 0.23 | **0.01** | 0.21 | **0.02** | 0.13 | 0.16 |
| **Triglycerides** | 0.01 | 0.90 | 0.08 | 0.40 | -0.01 | 0.88 |
| **Cholesterol** | 0.05 | 0.59 | 0.19 | **0.03** | -0.01 | 0.91 |
| **LDL** | 0.08 | 0.40 | 0.23 | **0.01** | -0.01 | 0.93 |
| **HDL** | -0.01 | 0.89 | -0.06 | 0.51 | -0.01 | 0.89 |
| **ALT** | 0.02 | 0.79 | 0.06 | 0.51 | -0.02 | 0.81 |
| **AST** | 0.03 | 0.70 | 0.05 | 0.56 | 0.04 | 0.63 |
| **GGT** | 0.17 | 0.05 | 0.18 | **0.04** | 0.02 | 0.83 |
| **CRP** | 0.16 | 0.07 | 0.12 | 0.18 | -0.06 | 0.51 |
| **TNF** | 0.16 | 0.07 | 0.05 | 0.60 | 0.01 | 0.88 |
| **IFN** | 0.13 | 0.16 | 0.00 | 0.97 | 0.02 | 0.83 |
| **IL6** | 0.16 | 0.07 | 0.13 | 0.15 | 0.09 | 0.32 |
| **IL8** | 0.01 | 0.87 | -0.02 | 0.85 | -0.05 | 0.57 |

Abbreviations: ALT, alanine transaminase; AST, aspartame transaminase; BMI, body mass index; CRP, C-reactive protein; GGT, gamma-glutamyl transpeptidase; HDL, high-density lipoprotein; HOMA-IR: homeostatic model assessment for insulin resistance; IFNγ, interferon gamma; IGF-1, insulin-like growth factor 1; IL-6, interleukin 6; IL-8, interleukin 8; LDL, low-density lipoprotein; SAT, subcutaneous adipose tissue; TNFα, tumor necrosis factor-α; VAT, visceral adipose tissue. *Adiponectin was not measured at week 50.
